# Successful Surgical Operation

**Published:** 1847-10

**Authors:** 


*Successful Surgical Operation.*—A paper in New Hampshire states that an eminent physician of Manchester recently opened the stomach of a patient, and removed several hard substances which had completely obstructed the passage from it. The patient is doing well, and will undoubtedly recover. The operation was performed in the presence of several gentlemen, and occupied from ten to fifteen minutes. A more full report will doubtless be made of this operation, if, indeed, it turns out that there was anything remarkable in it.—*Ibid.*

